# An epigenetic association analysis of childhood trauma in psychosis reveals possible overlap with methylation changes associated with PTSD

**DOI:** 10.1038/s41398-022-01936-8

**Published:** 2022-04-30

**Authors:** Solveig Løkhammer, Anne-Kristin Stavrum, Tatiana Polushina, Monica Aas, Akiah A. Ottesen, Ole A. Andreassen, Ingrid Melle, Stephanie Le Hellard

**Affiliations:** 1 NORMENT, Department of Clinical Science, University of Bergen, Bergen, Norway; 2 Dr. Einar Martens Research Group for Biological Psychiatry, Center for Medical Genetics and Molecular Medicine, Haukeland University Hospital, Bergen, Norway; 3 NORMENT, Division of Mental Health and Addiction, Oslo University Hospital & Institute of Clinical Medicine, University of Oslo, Oslo, Norway; 4 Department of Mental Health Research and Development, Division of Mental Health and Addiction, Vestre Viken Hospital Trust, Oslo, Norway; 5 Norwegian Centre for Violence and Traumatic Stress Studies, Oslo, Norway; 6 Bergen Center for Brain Plasticity, Haukeland University Hospital, Bergen, Norway

**Keywords:** Epigenetics in the nervous system, Psychiatric disorders

## Abstract

Patients with a severe mental disorder report significantly higher levels of childhood trauma (CT) than healthy individuals. Studies have suggested that CT may affect brain plasticity through epigenetic mechanisms and contribute to developing various psychiatric disorders. We performed a blood-based epigenome-wide association study using the Childhood Trauma Questionnaire-short form in 602 patients with a current severe mental illness, investigating DNA methylation association separately for five trauma subtypes and the total trauma score. The median trauma score was set as the predefined cutoff for determining whether the trauma was present or not. Additionally, we compared our genome-wide results with methylation probes annotated to candidate genes previously associated with CT. Of the patients, 83.2% reported CT above the cutoff in one or more trauma subtypes, and emotional neglect was the trauma subtype most frequently reported. We identified one significant differently methylated position associated with the gene *TANGO6* for physical neglect. Seventeen differentially methylated regions (DMRs) were associated with different trauma categories. Several of these DMRs were annotated to genes previously associated with neuropsychiatric disorders such as post-traumatic stress disorder and cognitive impairments. Our results support a biomolecular association between CT and severe mental disorders. Genes that were previously identified as differentially methylated in CT-exposed subjects with and without psychosis did not show methylation differences in our analysis. We discuss this inconsistency, the relevance of our findings, and the limitations of our study.

## Introduction

Childhood trauma (CT) is a well-established risk factor for developing a spectrum of severe mental disorders throughout life [1, 2], especially psychotic symptoms [3, 4]. Among patients with severe mental illness, reported prevalences of sexual and physical abuse are 26 and 39% [5], respectively, compared to 11 and 7% in healthy controls [6]. CT has diverse effects: children can show increased vulnerability to stressful events or resilience against adverse experiences [7, 8]. Both pre-clinical [9, 10] and clinical studies [8, 11, 12] suggest that adverse environmental risk factors, e.g., abuse and neglect, facilitate psychopathological processes in the brain during sensitive developmental periods. Over time, the accumulation of such environmental factors could modify distinct tissues and/or cell lineages and increase the risk of developing a psychiatric disease.

DNA methylation is an epigenetic mechanism that dynamically regulates gene expression by adjusting DNA accessibility to the transcriptional machinery. Environmental factors can cause long-lasting, altered DNA methylation patterns [13, 14]. DNA methylation may “embed” environmental factors in our genome, thus linking CT and stress-related neuropsychiatric disorders [15]. In rodent models, distress and adversities during development can induce DNA methylation changes in the brain, which can persist in adulthood and might even be transmitted through generations [16]. Several studies have aimed to replicate such findings in humans. However, heterogeneity in study designs (methylation platforms, tissues investigated, statistical methods, trauma definitions, study populations) complicates the direct comparison of findings and the interpretation of results [17]. A systematic review, looking at DNA methylation association to CT in individuals with and without a history of a psychotic episode, suggested *BDNF*, *GCH1*, *MPB*, *NDEL1*, *AKT1*, *DICER1*, *DROSHA*, *COMT*, *DISC1*, *SLC6A4*, *NR3C1*, *KITLG*, *FKBP5*, *OXTR*, *IL-6*, *TNFa, IL1a, IL1B, IL8*, and *PTGS* as candidate genes [18]. Presumably, epigenetic regulation of some of these genes might be involved in the psychopathology of severe mental disorders, while others may be vulnerability or resilience factors.

Large blood-based epigenome-wide association studies (EWAS) have been conducted for depression [19] and post-traumatic stress disorder (PTSD) [20]. Although the association between CT and DNA methylation has been widely studied (see review [21]), predominantly in healthy samples [22–25], no known research has investigated genome-wide DNA methylation associated with CT in severe mental disorders. Only one study investigated the association between CT and DNA methylation in first-episode schizophrenia by analysing specific cell lines. It found lower DNA methylation levels in patients with a history of CT [26]. Therefore, little is known of the epigenetic marks associated with CT in psychiatric illnesses. We now report an EWAS on 602 patients diagnosed with schizophrenia, bipolar disorder, or another psychotic disorder and have responded to the Childhood Trauma Questionnaire-short form (CTQ-SF). We aim to identify modulations in DNA methylation associated with CT in severe mental disorders.

## Materials and methods

### Sample

We included patients from the NORMENT study, also called the Thematically Organized Psychosis (TOP) study, Oslo (Norway). Participants were enrolled in the study between 2007 and 2018. A further description of the NORMENT sample has been published previously [27]. For our EWAS, we included 602 patients (no healthy controls). Previous studies have included smaller or equal samples sizes and detected moderate effects [22, 24]. In the lack of a standardized way to estimate the effect size of EWAS case studies (contrary to EWAS case-control studies), we considered our sample of 602 patients would provide enough power to detect moderate effects.

### Patients

Patients were of European ancestry aged between 18 and 64 years (median = 28.0 years). All patients included have a DSM-IV diagnosis for a severe mental disorder based on a structured diagnostic interview (SCID-I for DSM-IV), which included the following: schizophrenia group (*n* = 268) [schizophrenia (*n* = 192), schizophreniform disorder (*n* = 26), schizoaffective disorder (*n* = 50)], bipolar disorder group (*n* = 229) [bipolar disorder I (*n* = 150), bipolar disorder II (*n* = 63), bipolar not otherwise specified (*n* = 16)] and the other psychosis group (*n* = 105) [psychotic disorder not otherwise specified (*n* = 55), major depressive disorder (*n* = 23), delusional disorder (*n* = 18), brief psychotic disorder (*n* = 9)]. All patients were outpatients or stable inpatients from psychiatric units. Previous studies have described patients' exclusion criteria and the clinical assessments of patients [27]. All patients were assessed with the five-factorial model of the Positive and Negative Syndrome Scale (PANSS) [28], the Inventory of Depressive Symptomatology (IDS) [29], and the Global Functioning Scale divided into function (GFS-F) and symptoms (GFS-S) [30]. A fasting blood sample was drawn in the morning within a narrow time range. All participants gave written informed consent. The Regional Committee for Medical Research Ethics and The Norwegian Data Inspectorate approved the study with ethical approval #2009/2485, #2013/1727.

### Psychosis- and no-psychosis patient groups

To evaluate the association between DNA methylation and psychotic symptoms we divided patients into two groups, 514 with a history of psychosis and 81 (all with bipolar disorder, BP) negative for psychosis. Seven patients with no information on a history of psychosis were excluded. The analytic pipeline was run for psychosis/no psychosis separately, in addition to all 602 patients together. For further details, see the Supplementary Methods.

### Childhood trauma questionnaire-short form (CTQ-SF)

Childhood trauma was retrospectively reported using the CTQ-SF (Norwegian version) [31, 32]. This 28-item self-report questionnaire includes five subtypes of trauma (emotional abuse, physical abuse, sexual abuse, emotional neglect, and physical neglect) and a total score of “all trauma”. We set a predefined cut-off score by the median per subtype and total score, and thereby a score above the median was defined as trauma. This cut-off is consistent with previous research [33]. Additionally, we report the percentage of individuals with scores above this cut-off. For further details, see the Supplementary Methods.

### Imputation

From CTQ-SF data, we imputed missing values from participants with less than eight missing values in total, or less than two missing values per trauma subtype. Imputation was performed based on gender and the median value of the total score per sub-score. If more than two missing values were within one trauma subtype, but with not more than eight missing values in total, this domain was removed for the individual, and the rest kept. One participant did not meet this pre-established criteria and was removed. Of the remaining 602 participants, *n* = 31 (5.1%) had one or more imputed values from the CTQ-SF.

### DNA methylation quantification

Methylation quantification was completed using the Illumina Infinium^®^ Methylation EPIC BeadChip (Illumina, Inc. San Diego, USA).

### Pre-processing and quality control

Samples were imported to the statistical programming software R (version 3.6.2). The Bioconductor R-package *meffil* [34] was used for quality assessment and data pre-processing. Samples were typed for methylation status in three separate batches. Initial principal component analysis (PCA) of the unprocessed data showed that these batches had a significant effect on the data; hence, the pre-processing steps were carried out separately for each batch before merging the samples into one large dataset. Samples and CpG sites that failed quality control checks were removed (see Supplementary Methods). Finally, samples were normalized using functional normalization. Based on plots generated by the *meffil.plot.pc.fit* function in *meffil*, a visual evaluation of residual variation by the principal components (PCs) was used to decide the number of PCs for normalization. Twenty PCs were included to normalize the first and second typing batches and 25 were included for the third batch.

The samples from the three batches were combined, and *ComBat* [35] was used to remove the batch effect from the typing. Technical replicates included in the different typing batches were used to evaluate whether the datasets could be merged. After visually inspecting a PCA plot (Supplementary Fig. 1), we concluded that the quality of data pre-processing was satisfactory, and the data was merged without further normalization. The samples from individuals who answered the CTQ-SF were extracted. The final dataset had 602 samples and 759,742 probes.

### Statistical analysis

For the 602 patients, we applied a linear regression model where DNA methylation values were regressed against trauma scores to seek differentially methylated positions (DMPs). Six distinct trauma scores from the CTQ-SF—the total trauma score (“all trauma”) and the five trauma subtypes—were analysed separately. Because of the high correlation between the trauma subtypes, we did not further correct for the number of trauma subtypes tested. After testing three regression models (see Supplementary Methods), we selected Model A since it shows the best quantile-quantile plot (Q-Q plot). Model A includes trauma score, age, sex, smoking, and the five first PCs as covariates. Scree plots, Q-Q plots, and correlations of PCs with biological and technical covariates are shown in Supplementary Figs. 2–4.

We used *limma* to perform linear regression and thereby identification of DMPs [36], the *comb-p* algorithm to identify differentially methylated regions (DMRs) [37], and *stats* [38] for PCA. As recommended, M-values were applied for statistical analysis [39]. DMPs and DMRs were annotated to genes if CpGs were located in promoter regions, 5′/3′ UTRs, or in the gene itself through Illumina annotations [40]. For DMPs, nominal *p* values were converted to false discovery rate (FDR) values following the Benjamini and Hochberg approach [41]. For DMRs, Šidák correction at the 1% level was set as the multiple testing correction. Consistent with previous studies, a significance threshold was set to FDR <0.05 or Šidák *p* < 0.05 [14]. Previous studies have demonstrated that in the absence of a reference correlation map such as a linkage disequilibrium map, Šidák correction can account for some of the correlation being less conservative than Bonferroni correction [42]. Thus, in the absence of a correlation map reference as such in EWAS, we applied Šidák correction for DMRs rather than FDR correction as it assumes independence between all probes.

### Gene pathway analysis

Through *missMethyl* [43], we performed a gene pathway analysis to identify possible mechanisms of genes identified in our EWAS.

### Investigation of the association between CT and DNA methylation in candidate genes

Using the Bioconductor annotation package for Illumina annotations (reference genome hg19) [40], 1678 methylation probes were annotated to previously published candidate genes. We looked up the *p* values for these probes in the EWAS result lists (see Supplementary Dataset 1). Probes with *p* value < 2.98E-05 (0.05/1678) were taken to be significantly associated with CT.

## Results

### Childhood trauma data

After imputation, we analysed 602 patients with CTQ-SF data (Table 1). A total of 83.2% reported a trauma score over the pre-set cutoff in one or more trauma traits, and emotional neglect was the most frequently reported trauma. The median CTQ-SF total score for all trauma types was 39. The CT distribution was similar in the psychosis- and non-psychosis groups (Supplementary Fig. 5); thus, we merged these groups. The age distribution in the patient group reporting trauma and the non-trauma group was similar (Supplementary Fig. 6). We concluded that age would not be a confounding factor for further analysis when merging these groups to a patient group of *n* = 602. Supplementary Figs. 7,8 show score distributions for each trauma trait and trauma distributions for each diagnostic group.

**Table 1:** Socio-demographic characteristics and clinical features for patients.

Caracteristic	SCZ (*n* = 268)	BP (*n* = 229)	Other (*n* = 105)	Total (*n* = 602)
Age, mean ± SD	30.0 ± 9.8	32.8 ± 11.5	28.7 ± 9.9	30.8 ± 10.6
Sex, *n* (%)				
Male	149 (55.6)	91 (39.7)	60 (57.1)	300 (49.8)
Female	119 (44.4)	138 (60.3)	45 (42.9)	302 (50.2)
Smokers, *n* (%)				
Male	94 (63.1)	57 (62.6)	33 (55.0)	181 (60.3)
Female	73 (61.3)	66 (47.8)	24 (53.3)	163 (54.0)
Medication, *n* (%)				
≥one type of antipsychotic	228 (85.1)	131 (57.2)	70 (66.7)	429 (71.3)
≥one type of antidepressant	76 (28.4)	76 (33.2)	33 (31.4)	185 (30.7)
≥one type of antiepileptic	38 (14.2)	85 (37.1)	9 (8.6)	132 (21.9)
Lithium	52 (19.4)	46 (20.1)	-	98 (16.3)
Clinical assessment				
PANSS score, mean ± SD	65.7 ± 16.3	45.7 ± 9.8	54.7 ± 14.7	56.1 ± 13.4
GFS-S score, mean ± SD	41.7 ± 11.9	56.6 ± 11.8	49.0 ± 13.2	48.7 ± 13.8
GFS-F score, mean ± SD	42.2 ± 10.5	54.4 ± 13.1	52.4 ± 13.6	48.6 ± 13.4
IDS score, mean ± SD	18.1 ± 11.2	17.6 ± 11.2	18.1 ± 11.3	17.9 ± 11.2
CTQ-SF score				
Sexual abuse, median (min-max)	5 (5–25)	5 (5–25)	5 (5–25)	5 (5–25)
Emotional abuse, median (min-max)	9.5 (5–25)	9 (5–25)	9 (5–24)	9 (5–25)
Emotional neglect, median (min-max)	12 (5–25)	11 (5–25)	11 (5–24)	11 (5–25)
Physical abuse, median (min-max)	5 (5–25)	5 (5–25)	5 (5–15)	5 (5–25)
Physical neglect, median (min-max)	7 (5–19)	6 (5–22)	7 (5–16)	7 (5–22)
Total trauma, median (min-max)	40 (25–117)	37 (25–120)	39 (25–86)	39 (25–120)

Overview of socio-demographic characteristics and clinical features in patients categorized by diagnosis and in the total patient group. The percentage of people who smoke tobacco is calculated by frequencies for males and females separately. Childhood trauma prevalences are given for each trauma trait by the median score, which is also the study’s predefined cutoff for trauma.

*SCZ* schizophrenia, *BP* bipolar disorder, *Other* another psychotic disorder, *PANSS* positive and negative syndrome scale, *GFS-F* global functioning scale, functioning, *GFS-S* global functioning scale, symptoms, *IDS* inventory of depressive symptomatology, *CTQ-SF* childhood trauma questionnaire-short form.

### Genome-wide identification of differentially methylated positions

**Fig. 1:**
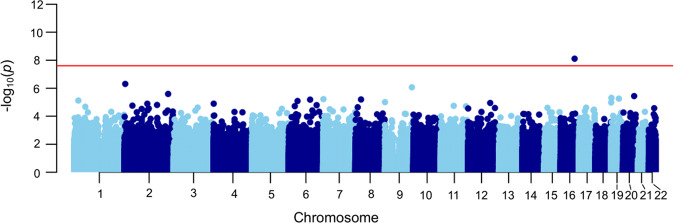
Manhattan plot for physical neglect in all patients (*n* = 602) obtained from the Childhood Trauma Questionnaire, Short-Form (CTQ-SF). The Manhattan plot illustrates associations between methylation probes and physical neglect in a group of patients with a severe mental disorder. All chromosomes (except sex chromosomes) are displayed on the x-axis, while *p* values (negative tenfold scale) are displayed on the y-axis. The red line on the y-axis indicates the pre-set cutoff for significance. The methylation probe cg07625619 on chromosome 16 survived multiple testing (% DNA methylation difference = 2.54, *p* value = 7.74E-09, FDR = 0.0059).

We applied six separate EWASs for the six trauma traits (five subtypes and total score) to the 602 patients. After multiple testing correction, one significant DMP (cg07625619) remained, which was associated with physical neglect (% DNA methylation difference = 2.54, *p* value = 7.74E-09, FDR = 0.0059). Figure 1 illustrates the genome-wide findings for physical neglect and Table 2 presents the 20 most significant DMPs for physical neglect. The same DMP was also significant when narrowing the analysis to the 514 patients with a history of psychosis (% DNA methylation difference = 2.67, *p* value = 3.25E-08, FDR = 0.025). This DMP was associated, at FDR < 0.1, with all trauma in the 602 patients (% DNA methylation difference = 2.14, *p* value = 1.02E-07, FDR = 0.077), and in the patients with psychosis (% DNA methylation difference = 2.15, *p* value = 8.78E-08, FDR = 0.067). For complete results, see Supplementary Dataset 1. No other single probes were associated at the FDR < 0.05 level for the other trauma traits.

**Table 2:** The 20 most significant DMPs associated with physical neglect for 602 patients with a severe mental disorder.

Probe	DNA methylation difference (%)	*p* value	FDR value	Chr	Position	Gene annotation
cg07625619	2.54	7.74E-09	0.0059	16	69050940	*TMCO7* (Body)
cg25532061	4.27	4.91E-07	0.19	2	3622930	*RPS7(*1stExon;5′UTR)
cg13928649	6.87	8.67E-07	0.22	9	133540341	*PRDM12* (Body)
cg05691168	3.22	2.57E-06	0.49	2	216176809	*ATIC* (1stExon;5′UTR)
cg19739407	−2.61	3.63E-06	0.50	20	55200749	
cg03156477	2.44	4.89E-06	0.50	19	1924758	*SCAMP4* (3′UTR)
cg24900663	4.04	5.56E-06	0.50	19	38810166	*KCNK6* (TSS1500)
cg18893098	−2.77	6.00E-06	0.50	7	1136568	*C7orf50* (Body)
cg27336360	−3.72	6.40E-06	0.50	8	28174350	*PNOC* (TSS1500)
cg08700776	1.56	6.59E-06	0.50	6	106416368	
cg08898653	5.56	7.64E-06	0.52	1	20811220	*CAMK2N1* (Body)
cg20253785	3.38	8.21E-06	0.52	6	44043009	*LOC101929705* (TSS1500)
cg20229853	5.37	9.86E-06	0.57	9	214915	*C9orf66;DOCK8* (1stExon;5′UTR)
cg19447984	3.44	1.05E-05	0.57	19	897424	*C19orf22* (3′UTR)
cg03753241	4.42	1.13E-05	0.57	12	109915367	*KCTD10;UBE3B* (TSS1500;TSS200)
cg18695931	2.42	1.26E-05	0.57	4	887295	*GAK* (Body)
cg00546774	5.27	1.28E-05	0.57	2	112811810	*TMEM87B* (TSS1500)
cg00335252	3.94	1.57E-05	0.65	2	161245200	*RBMS1* (Body)
cg13619723	−2.88	1.62E-05	0.65	6	138548861	*ARFGEF3* (Body)
cg09501518	3.51	1.77E-05	0.65	2	39004204	*GEMIN6* (TSS1500)

DNA methylation difference (%) is calculated by (log fold change – 1) × 100%. Genomic annotation is obtained from Illumina reference lists.

### Identification of differentially methylated regions (DMRs)

DMRs accumulate the differences in methylation of consecutive probes in a region and can therefore capture the additional signal from single positions. For the six trauma traits, we identified seventeen DMRs at the significance level Šidák *p* < 0.05 (Table 3). Twelve of them were annotated to genes through Illumina annotations [40] according to hg19. The *HM13; PSIMCT-1* genes were shared between two traits (sexual abuse and all trauma), while the remaining genes were unique to one trait.

**Table 3:** Differentially methylated regions for different trauma traits significant at the 0.05 level.

Trauma traits	Chr	Start	End	*N* probes	*p* value	Šidák *p*	Gene annotation
Physical abuse	6	29648161	29648757	18	4.31E-15	4.21E-17	
Sexual abuse	6	33048086	33048880	17	7.10E-10	2.36E-15	
Sexual abuse	20	30134929	30135363	8	3.67E-09	1.23E-10	*HM13;PSIMCT-1* (Body;TSS200;TSS1500)
Sexual abuse	17	81060149	81060260	3	3.36E-06	7.25E-07	
Physical neglect	21	45705543	45705743	8	1.48E-05	7.41E-07	*AIRE* (TSS200)
Physical neglect	17	6899207	6899578	9	3.78E-06	1.58E-06	*ALOX12* (TSS200;Body;1stExon)
Physical neglect	7	28452066	28452290	5	1.94E-05	1.39E-06	*CREB5* (1stExon;5′UTR;TSS200)
Emotional abuse	10	133558786	133558972	3	6.30E-04	1.12E-05	
Emotional abuse	12	52462839	52463051	4	6.30E-04	4.03E-05	*ATG101* (TSS1500)
Emotional abuse	6	28583971	28584156	10	6.30E-04	5.79E-05	
Physical neglect	17	78851149	78851263	3	3.40E-04	1.01E-04	*RPTOR* (Body)
Physical neglect	8	11666485	11666695	7	6.15E-05	1.44E-04	*FDFT1* (TSS200;Body;1stExon)
Physical abuse	19	3480508	3480673	5	8.16E-04	1.53E-04	*SMIM24;C19orf77* (TSS200;5′UTR;1stExon)
Physical neglect	9	214690	214916	2	1.70E-04	2.27E-04	*C9orf66;DOCK8* (TSS200;1stExon;5′UTR)
Sexual abuse	1	205819251	205819493	7	3.01E-05	5.14E-04	*PM20D1* (TSS200;TSS1500;5′UTR;1stExon)
Physical neglect	6	150346816	150347013	6	3.70E-04	0.00279	*RAET1L* (TSS1500;TSS200)
All trauma	20	30135144	30135144	4	5.63E-04	0.0168	*HM13;PSIMCT-1* (Body;TSS200)

Šidák *p* values represent *p* values after multiple testing corrections. DNA methylation difference (%) is calculated by (log fold change – 1) × 100%. Genomic annotation is obtained from Illumina reference lists.

### Gene pathway analysis

The gene pathway analysis revealed no significant findings. This negative result was expected, as a limitation of the investigation was the low number of genes included.

### Investigation of the association between CT and DNA methylation in candidate genes

A previous review listed 20 candidate genes associated with CT in patients with psychotic features and healthy subjects: *BDNF*, *GCH1*, *MPB*, *NDEL1*, *AKT1*, *DICER1*, *DROSHA*, *COMT*, *DISC1,* *SLC6A4*, *NR3C1*, *KITLG*, *FKBP5*, *OXTR*, *IL-6*, *TNFa*, *IL1a*, *IL1B*, *IL8*, and *PTGS* [18]. To investigate the association of single CpGs located within or near these genes, we annotated 1678 methylation probes to the genes through Ilumina annotations (hg19) [40], thus setting the experiment-wide significance threshold to 0.05/1678 = 2.98E-05.

We then investigated the genetic overlap with our results for all 602 patients, followed by the 514 patients with a history of psychosis for five trauma subtypes and total trauma score. None of the CpGs annotated to the candidate genes was associated with CT, i.e., no CpG had a *p* value < 2.98E-05.

## Discussion

We performed an EWAS in 602 patients with a severe mental disorder for five trauma subtypes and a summative trauma score. One DMP was significantly associated with physical neglect in all patients, both with and without psychosis. For the remaining CT traits, we found no significantly associated DNA methylation sites with FDR <0.05. Analysis revealed seventeen DMRs (Šidák *p* < 0.05) associated with all trauma, sexual abuse, emotional abuse, physical abuse, and physical neglect. We identified no significant association to methylation sites located within or near candidate genes previously reported to be CT-associated in healthy individuals or patients.

The significant CpG (cg07625619) associated with physical neglect is located in the body of the gene *TANGO6* (transport and Golgi organization protein 6 homolog), also called *TMCO7* (transmembrane and coiled-coil domain-containing protein 7) (reference genome GRCHh37/hg19 [44]). *TANGO6* encodes a known interactor of MACF1 (microtubule-actin crosslinking factor 1). MACF1 plays a major role in neural progenitor proliferation and neural migration through dynamic regulation of the cytoskeleton [45]. Neural migration is critical for constructing neuronal connections in brain development [46]. MACF1 interacts with the DISC1 (disrupted-in-schizophrenia 1) protein, reported as linked with psychosis [46, 47]. A large-scale GWAS conditioned on *DISC1* found eight genes associated with susceptibility to psychosis, among them *TANGO6* [48]. This is interesting in the context of repeated reports of synaptic pathophysiology in patients with schizophrenia [49, 50]. Further, differential expression of *TANGO6* was reported in a mouse model of cognitive dysfunction [51]. However, no known studies have previously reported *TANGO6* linked to trauma-related psychopathology. Additionally, yet not significant, we identified several interesting DMPs associated with physical neglect, including the *PNOC* (prepronoceptin) gene, previously found implicated in PTSD [52].

**Fig. 2:**
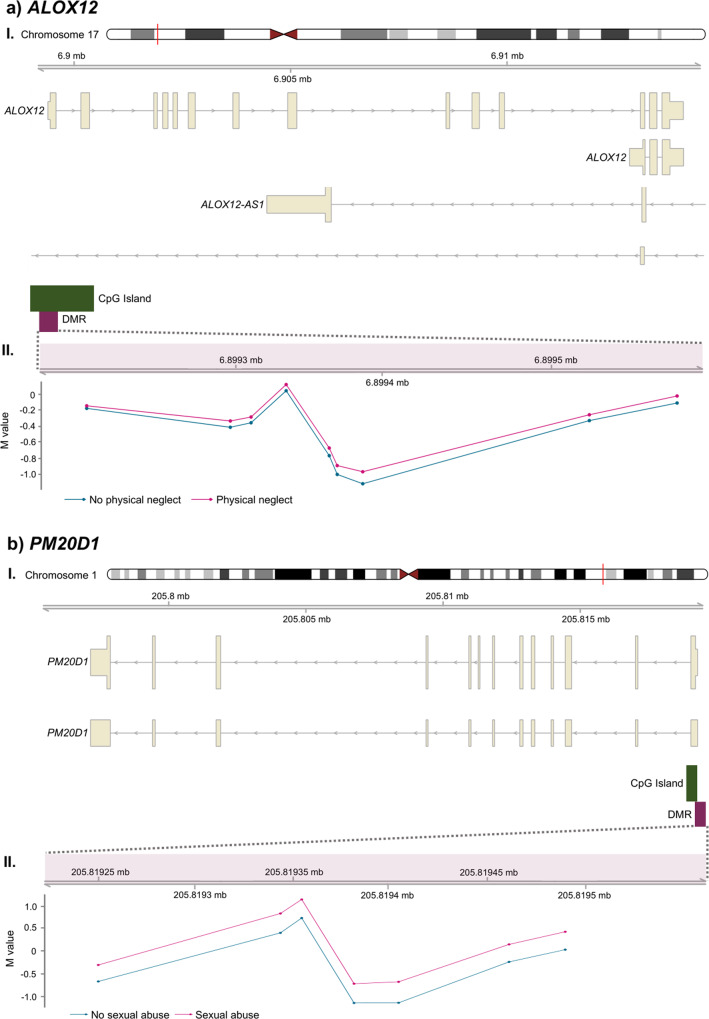
Differentially methylated regions (DMRs) related to the genes ALOX12 and PM20D1. **a**, **b** The DMRs for ALOX12 and PM20D1, respectively. For each DMR, panel I reports the genomic location, gene organisation and location of the DMR relative to the gene and CpG Island. Panel II illustrates average methylation M-values for CpGs included in the DMR. Each CpG is represented by a dot. Purple represents the average methylation in individuals exposed to trauma (above the trauma cut-off) and the blue the average M-values for individuals not exposed to trauma (below the trauma cut-off). The DMR located in ALOX12 was associated with physical neglect and included 9 CpG probes (*p*-value = 3.38E−06; Šidák *p*-value = 1.58E−06, a.II). The DMR located in PM20D1 was associated with sexual abuse and included 7 CpGs (*p*-value 3.01E−05; Šidák *p*-value 5.14E−04; b.II).

Several of the CT-associated DMRs were located near or in genes previously associated with severe mental disorders, cognitive impairment, and distress/trauma-related psychopathology. Two of the genes were previously linked to CT and PTSD Fig. 2 One of the physical neglect-associated DMRs is located in *ALOX12* (arachnoid acid 12 lipoxygenase), which is involved in oxidative stress regulation and is associated with PTSD in adults, including a reduction in the right prefrontal cortex thickness [53]. Another DMR, associated with sexual abuse, was found in *PM20D1* (peptidase M20 domain containing 1). Recently, a methylome-wide association study of saliva from 224 youths diagnosed with pediatric PTSD and a non-traumatized control group identified a DMR related to hypomethylation of *PM20D1* [54]. This was replicated in another cohort and related to gray matter volume in the right fusiform gyrus [54]. In military servicemen, a DMR locating to *PM20D1* was associated with longitudinal changes in PTSD symptoms [14]. A study investigating the relationship between CT and whole-blood methylation profiles in 45-year-old males with no specific health disorder found an association of *PM20D1* with childhood abuse in two independent cohorts [25]. Thus, *PM20D1* is associated with CT in older individuals with no specific phenotype, in youths and adults with PTSD, and, in our study, in CT-exposed adults with a severe mental disorder.

Further, we identified genes related to neurodevelopment and psychiatric disorders. One DMR associated with physical neglect is located near *C9orf66* (chromosome 9 open reading frame 66) and *DOCK8* (dedicator of cytokinesis 8). *DOCK8* is linked to altered neurodevelopment and intellectual disability [55, 56]. *DOCK8* copy number variants occur in patients with psychotic features [57] and *DOCK8* duplications were identified in patients with severe neuropsychiatric disorders, such as SCZ and BP, across five cohorts [58]. Another DMR associated with physical neglect was located within *CREB5* (cAMP-responsive element-binding protein 5) [59]. CREB proteins participate in synaptic plasticity, neurotransmission, neurodevelopment, dopamine receptor signal transduction, metabolism, and adaptive response to stress [59–61]. Studies using postmortem brain tissue from patients diagnosed with SCZ and BP indicate alterations in CREB protein and gene expression [62]. CREB is a known regulator of BDNF signal transduction [59, 60], and BDNF has repeatedly been suggested as a candidate gene for CT [18] and as a marker of active severe mental disorder regardless of the diagnostic entity [63]. Mice with a genetic susceptibility to major affective disorders have altered CREB activity in hippocampal tissue and, consequently, activation of BDNF and reduced resilience to acute restraint stress [60]. Furthermore, mice with hippocampal CREB deficiency show increased resilience to long-term stress and altered affective behaviors [64].

We report an association between physical neglect and a DMR located in the gene *RPTOR* (regulatory associated protein of MTOR complex 1), which was previously associated with neglect, sexual abuse, and physical abuse in a buccal tissue-based DNA methylation study of CT in a healthy but at high-risk sample [24]. *RPTOR* was reported as hypomethylated in SCZ case-controls across blood and brain tissue [65]. These findings indicate an association of CT with *RPTOR* in individuals with and without a severe mental disorder.

We also found that emotional abuse and physical neglect are associated with DMRs located to *ATG101* (autophagy-related 101) and *RAET1L* (retinoic acid early transcript L), respectively. *ATG101* was identified in a large-scale study of gene expression in the dorsolateral prefrontal cortex of SCZ patients and controls [66]. *RAET1L* was associated with SCZ in a case-control DNA methylation study [67]. Our study also found some associations with developmental stress. One DMR was located in the *PSIMCT-1* gene (*MCTS2P*) and the *HM13* gene, which is involved in genomic imprinting. *HM13* is essential for fetal development and is related to placental stress in pregnancy [68], and intrauterine growth restriction [69]. These relations indicate that the CT-associated epigenetic marks in severe mental disorders may also be related to stress *in utero*. Further studies are needed to investigate this association.

Since we investigated the association to CT in patients with a severe mental disorder, the observed DNA methylation alterations should be interpreted as possible epigenetic marks of CT in severe mental disorders. To our knowledge, no studies have investigated CT in such a group. Previous studies examining CT-DNA methylation associations have used heterogeneous study populations, including healthy individuals [22–25], first-episode psychosis [26], and PTSD phenotypes [54]. This makes it difficult to compare results. For example, we found no overlap between our results and a study investigating CT-associated DNA methylation patterns in an adult, healthy sample [22]. In patients, the identified CT-associated genetic regions could be involved in developing a psychosis phenotype or they may be regulated as a consequence of psychosis. When investigating healthy individuals with no psychosis phenotype, the identified epigenetic regions could (hypothetically) be involved in the genetic features of resilience. Genes involved in resilience do not necessarily have a protective effect on CT outcomes, but they may reduce the chances of adverse outcomes and may have an opposite effect on risk variants [70]. Ignoring these possible genetic hallmarks across study populations might lead to misleading interpretations of the effects of CT. Another important factor in CT is polyvictimization, which may limit the interpretation of results from individual trauma types.

In epigenetic research on neuropsychiatric disorders, it is critical to discuss whether blood is an adequate surrogate tissue for the brain. Significant differences in DNA methylation have been reported for schizophrenia [65, 71], PTSD [72], and autism [73] using blood samples. Research supports a high blood-brain concordance in DNA methylation levels [74]. In a study of SCZ patients, 94% of CpG-SNPs methylated in the brain overlapped with methylation in the blood [65]. However, some DNA methylation sites are variable in the blood and not in the brain, and vice versa [75]. Therefore, when investigating the effect of CT on brain phenotypes, we may possibly overlook disease-specific DNA methylation patterns when applying a blood-based EWAS.

A limitation of studying DNA methylation is its reversibility. CT has a spectrum of adverse health outcomes, which indicate systemic effects and give reasons to presume that there are stable DNA methylation changes in the blood. However, studies looking at cigarette smoking- and alcohol-associated DNA methylation patterns show that some DNA methylation changes are reversed in blood after the exposure ceases [76–78]. Peripheral blood frequently renews as opposed to brain cells—an essential difference between the two tissues since one of the mechanisms of reversing DNA methylation is thought to be dilution through cell division. Although white blood cells are post-mitotic [79], they are frequently renewed from the bone marrow. It is possible that CT-associated DNA methylation alterations might be reversed by the time adulthood is reached, affecting our results.

Our study reports DNA methylation changes at specific positions in several genomic regions associated with CT in severe mental disorders. These regions were located within genes observed to be dysregulated in mental illnesses like PTSD but also previously found to be associated with cognitive impairment and distress in utero. However, replication of our results is required in independent cohorts. We want to highlight the importance of carefully selecting the study design and methods when applying an EWAS to look at the effects of environmental factors, as previous studies in this field are highly heterogeneous. Further research should focus on a clear distinction between DNA methylation association for CT in healthy samples and affected individuals to separate genes involved in the psychopathology of severe mental disorders and genes playing a regulatory role in resilience mechanisms. Further research should also focus on the reversal of DNA methylation associated with trauma exposure and the timeframe for such reversal, which could be investigated using longitudinal data.

### Data deposition

Summary statistics can be provided upon request.

## Supplementary information

Supplementary Material

Supplementary Tables

## Data Availability

All code is available on request.
