# Supplementary material for: An epigenetic association analysis of childhood trauma in psychosis reveals possible overlap with methylation changes associated with PTSD
Source: Transl Psychiatry. 2022 Apr 30;12:177. doi: 10.1038/s41398-022-01936-8 (PMC9061740; doi:10.1038/s41398-022-01936-8)
Supplement: Supplementary file 1 — Supplementary Material [file 41398_2022_1936_MOESM1_ESM.pdf]

Supplementary Material for "An epigenetic association analysis of childhood trauma in psychosis reveals possible overlap with methylation changes associated with PTSD", Løkhammer et al.

I) Supplementary Methods

Psychosis- and no-psychosis patient groups – page 2
Childhood Trauma Questionnaire – short form (CTQ-SF) – page 2
Pre-processing and quality control – page 2
Linear regression models – page 3
References – page 4

II) Supplementary Figures

Supplementary Figure 1. Principal Component Analysis (PCA) plot for technical replicates – page 5
Supplementary Figure 2. Scree plot of the first ten Principal Components (PCs) – page 6
Supplementary Figure 3. Quantile-quantile plots (QQ-plots) from the three different models that were evaluated – page 7
Supplementary Figure 4. Correlation of the top ten Principal Components (PCs) with biological- and technical covariates – page 8
Supplementary Figure 5. Distribution of trauma scores in the psychosis (n=514) and no-psychosis (n=81) groups – page 9
Supplementary Figure 6. Age distribution in the trauma group (n=501) and non-trauma group (n=101) – page 10
Supplementary Figure 7. Trauma distribution for all patients (n=602) – page 11
Supplementary Figure 8. Distribution of trauma scores per diagnostic group – page 12

Psychosis- and no-psychosis patient groups

All patients had previously experienced psychosis except for 81 in the bipolar disorders (BP) group. To evaluate the possible association of psychosis with DNA methylation, we divided patients into two groups based on the previous experience of psychosis. This included 514 patients with a history of psychosis and 81 BP patients negative for psychosis. For seven patients, information on psychosis history was missing and they were excluded from this analysis. The analytic pipeline was run for psychosis/no psychosis separately, in addition to all 602 patients together.

Childhood Trauma Questionnaire-short form (CTQ-SF)

A history of childhood trauma was retrospectively reported using the Norwegian version of the Childhood Trauma Questionnaire-short form (CTQ-SF) developed by Bernstein et al. (1, 2). This 28-item self-report questionnaire includes five subtypes of trauma: emotional abuse, physical abuse, sexual abuse, emotional neglect and physical neglect, in addition to a total score of ‘all trauma’. The participants rate the twenty-five clinical items and three validity items on a five-point Likert scale ranging from 1 “never true” to 5 “very often true”. Seven out of the 28-items in the CTQ-SF are reversed: two items from physical neglect and five items from emotional neglect. Consistent with previous research (3), we set a predefined cut-off score by the median pr subtype and total score, meaning that a score above the median was defined as trauma. Previous studies have demonstrated the validity and reliability of the questionnaire, together with the considerable overlap between retrospectively reported trauma and trauma data obtained from other sources, independently of the psychopathology of psychosis (4, 5).

Pre-processing and quality control

The following samples and CpG sites were removed in our quality control procedure: (1) samples with 1% of sites, and sites with 1% of samples, with a detection p-value>0.01; (2) samples with a bead count<3 in 5% of the sites and sites with a bead count<3 in 5% of the samples; (3) sites targeted by probes that are known to have issues with cross-hybridization or contain single nucleotide polymorphisms (SNPs) that are close to target CpGs in the European population, following the approach of Zhou et al. (6); (4) samples with a mismatch between reported and predicted sex, as well as samples with an X-Y ratio standard deviation of more than five; (5) samples with

genotype mismatch; (6) probes from the sex chromosomes; and (7) samples from non-European individuals.

Statistical analysis – linear regression models

For linear regression, we tested three models including different numbers of covariates: Model A (Methylation ~ trauma score + age + sex + smoking status + PC1-PC5); Model B (Methylation ~ trauma score + age + sex + smoking + Cell Count Estimates + Plate) and Model C (Methylation ~ trauma score). In model A, the number of PCs was evaluated in a Scree Plot (Supplementary Figure 2). Q-Q-plots for all three models are shown in Supplementary Figure 3. Correlations between PCs and biological or technical covariates are illustrated in Supplementary Figure 4. Additionally to linear regression models, we evaluated the use of logistic analysis using trauma score as a dichotomous variable with the median score as cut-off. However, due to reduced power in a logistic model, we concluded to apply linear regression analysis only, using trauma score as a continuous variable.

References

1. Bernstein DP FL. Childhood Trauma Questionnaire: A retrospective self-report manual.San Antonio: Harcourt Brace and Company; 1998. 68 p.

2. Bernstein DP, Stein JA, Newcomb MD, Walker E, Pogge D, Ahluvalia T, et al. Development and validation of a brief screening version of the Childhood Trauma Questionnaire. Child Abuse & Neglect. 2003;27(2):169-90.

3. Aas M, Andreassen OA, Aminoff SR, Færden A, Romm KL, Nesvåg R, et al. A history of childhood trauma is associated with slower improvement rates: Findings from a one-year follow-up study of patients with a first-episode psychosis. BMC Psychiatry. 2016;16(1):126.

4. Fisher HL, Craig TK, Fearon P, Morgan K, Dazzan P, Lappin J, et al. Reliability and Comparability of Psychosis Patients' Retrospective Reports of Childhood Abuse. Schizophrenia Bulletin. 2009;37(3):546-53.

5. Liebschutz JM, Buchanan-Howland K, Chen CA, Frank DA, Richardson MA, Heeren TC, et al. Childhood Trauma Questionnaire (CTQ) correlations with prospective violence assessment in a longitudinal cohort. Psychol Assess. 2018;30(6):841-5.

6. Zhou W, Laird PW, Shen H. Comprehensive characterization, annotation and innovative use of Infinium DNA methylation BeadChip probes. Nucleic Acids Research. 2016;45(4):e22-e.

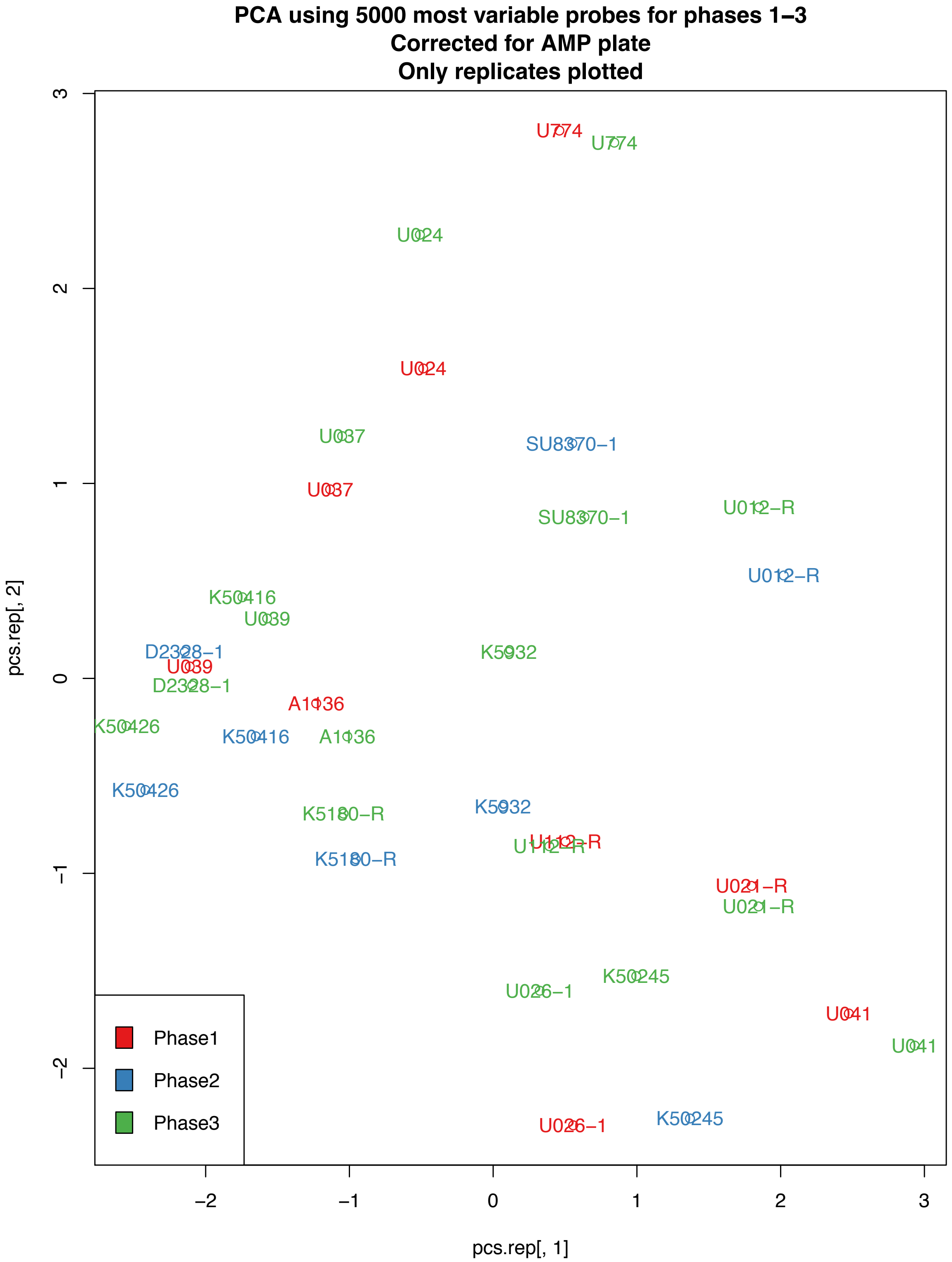

Supplementary Figure 1. Principal Component Analysis (PCA) plot for technical replicates. The samples included in the study were typed in 3 different batches. Some of the samples from the first batch were re-typed as technical replicates in the second or third batch in order to monitor batch effects. The plot comprises the 5000 most variable probes from the datasets' three phases, and only the technical replicates are plotted. The technical replicates appear near one another in the plot, suggesting that most of the batch effects were removed during pre-processing of the data.

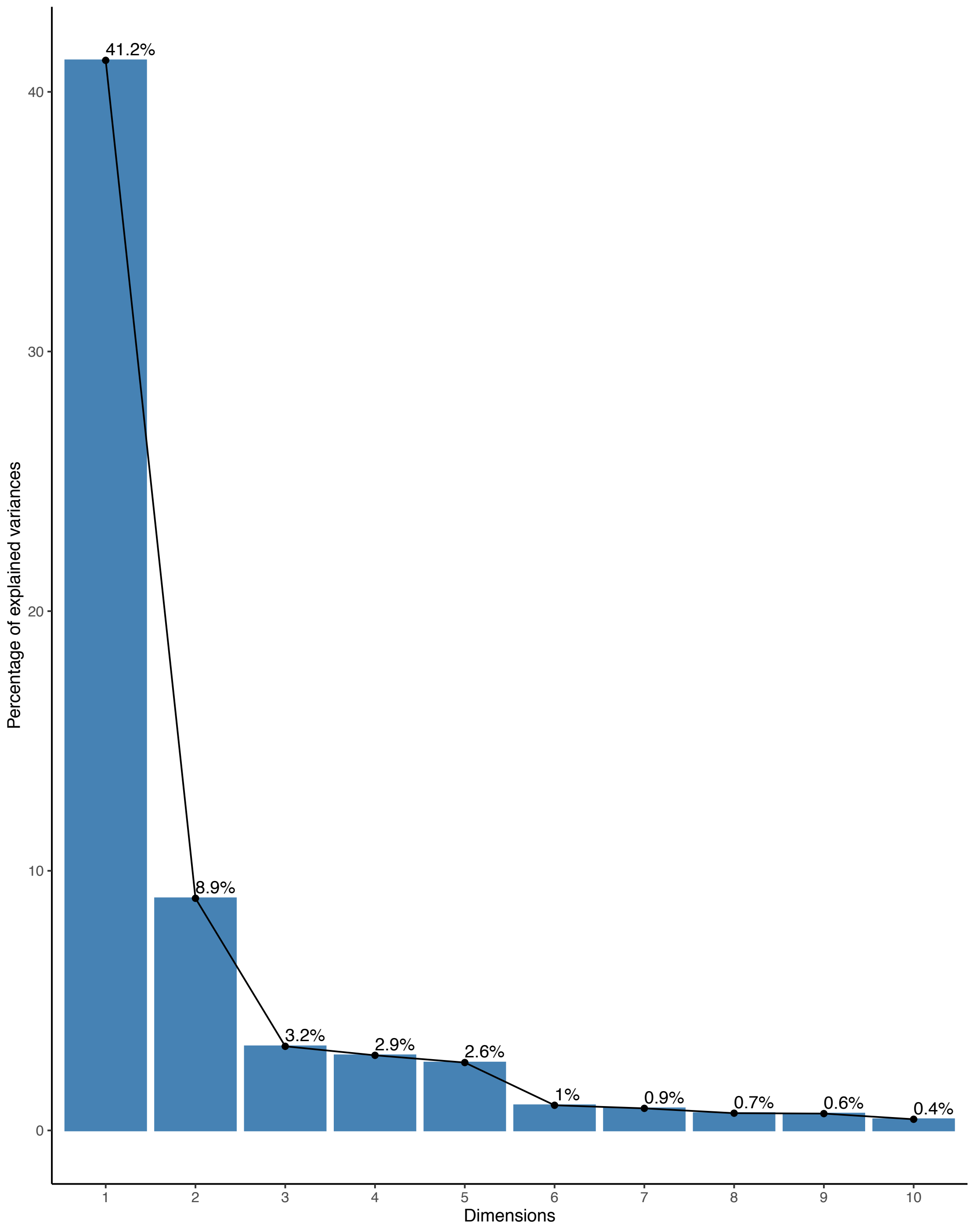

Supplementary Figure 2. Scree plot of the first ten Principal Components (PCs). The scree plot shows that the first five principal components, which we included in our final linear regression model as covariates, account for 58.8% of the total variance.

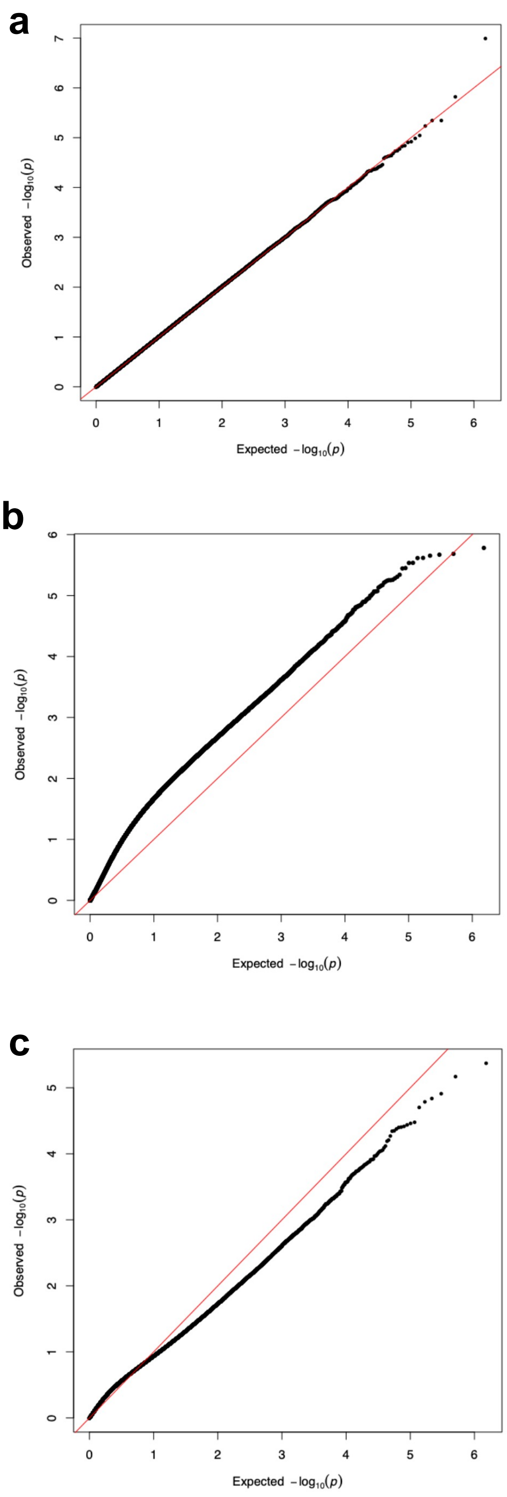

Supplementary Figure 3. Quantile-quantile plots (QQ-plots) from the three different models that were evaluated. For all QQ plots, the expected -log10 p-values on the x-axis are plotted against observed -log p-values on the y-axis, and the red line indicates the expected p-values under the normal distribution. Plot **(A)** illustrates the study's final model including age, sex, smoking status and PC1-PC5 as covariates, Plot **(B)** illustrates a model including age, sex, smoking status, Cell Count Estimates and Plate as covariates, Plot **(C)** illustrates a model including no covariates.

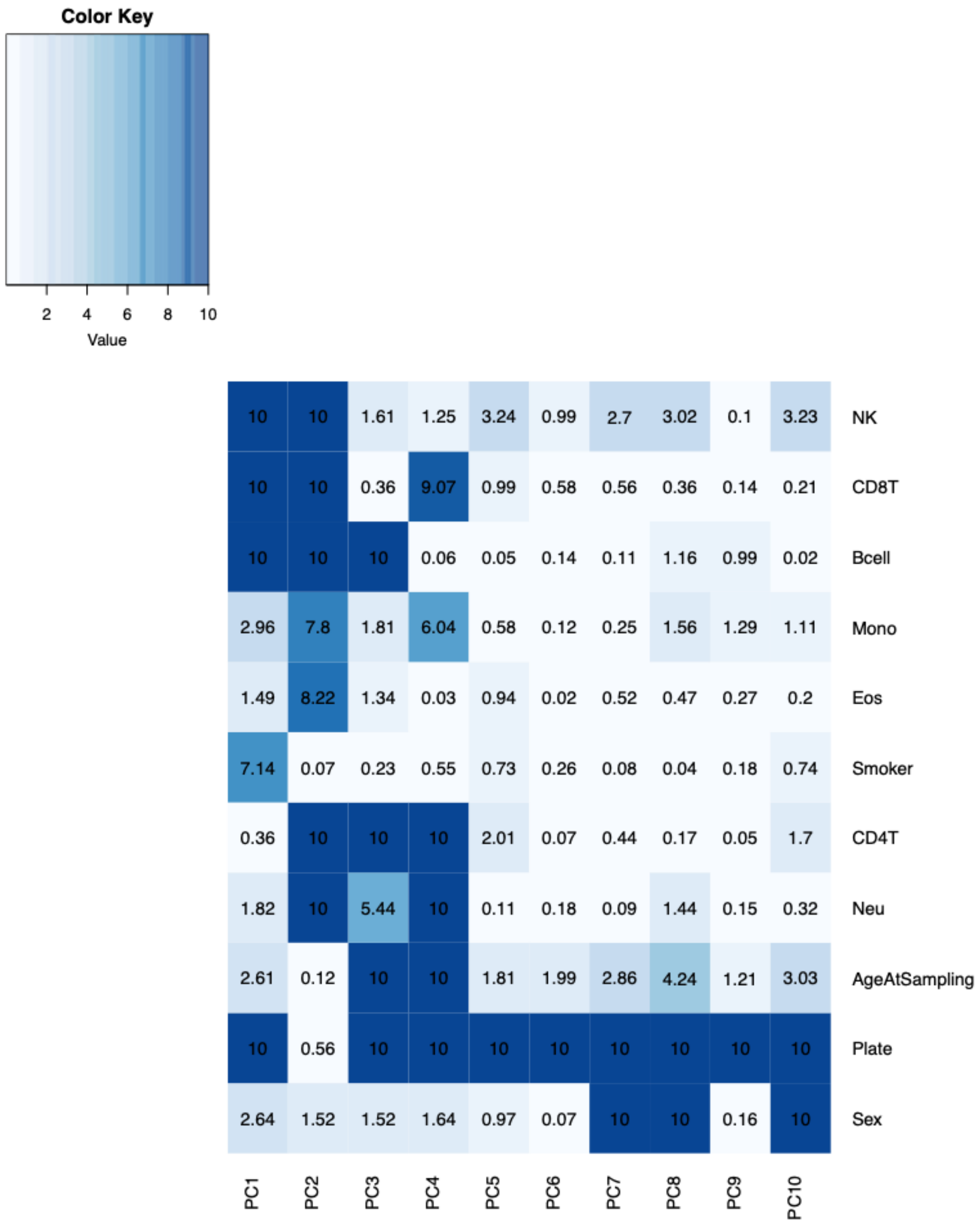

Supplementary Figure 4. Correlation of the top ten Principal Components (PCs) with biological- and technical covariates. The heatmap shows correlations, as the negative logarithm (base 10), between PC1-10 and known biological- and technical covariates. Biological covariates include sex, age (AgeAtSampling), smoking status (smoker) and cell-type proportions (NK, CD8T, Bcell, Mono, Eos, CD4T, Neu) estimated by the Houseman algorithm. Technical covariates include the array plate (Plate).

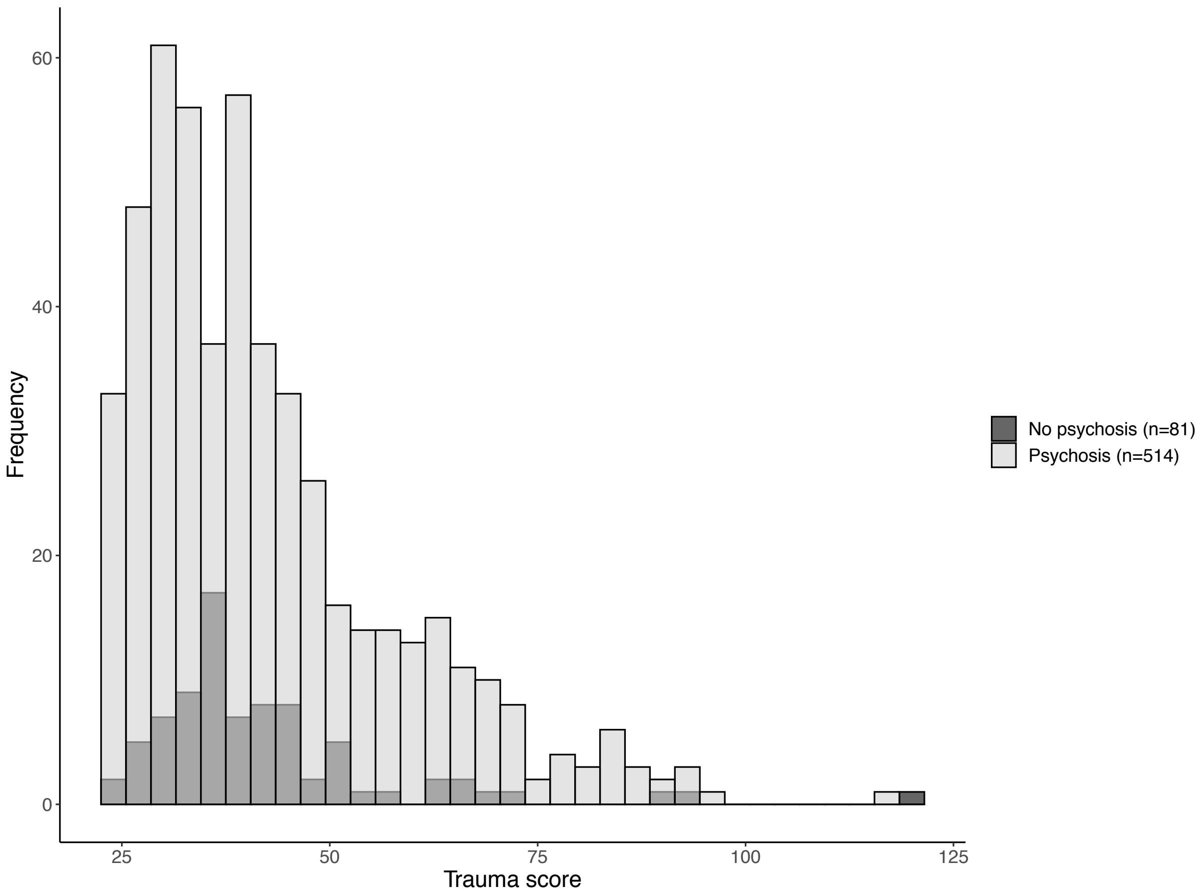

Supplementary Figure 5. Distribution of trauma scores in the psychosis (n=514) and no-psychosis (n=81) groups. The groups were dichotomized based on a reported history for one or more psychotic episodes. Trauma data was obtained from the Childhood Trauma Questionnaire, Short-Form (CTQ-SF). Trauma score is presented on the x-axis at a range between 25-120 where 120 is the highest possible score. Frequencies in the sample are presented on the y-axis.

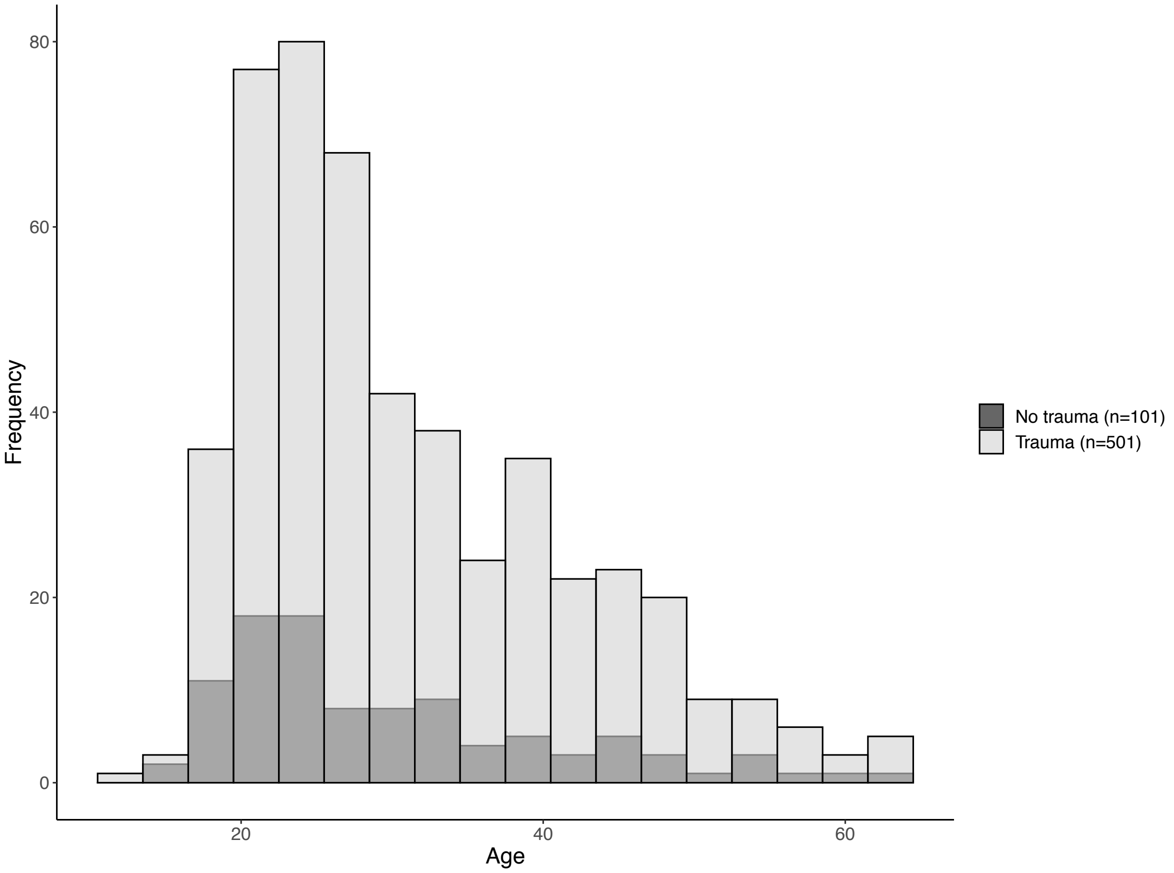

Supplementary Figure 6. Age distribution in the trauma group (n=501) and non-trauma group (n=101). The groups were dichotomized based on one or more reported trauma obtained from the Childhood Trauma Questionnaire, Short Form (CTQ-SF).

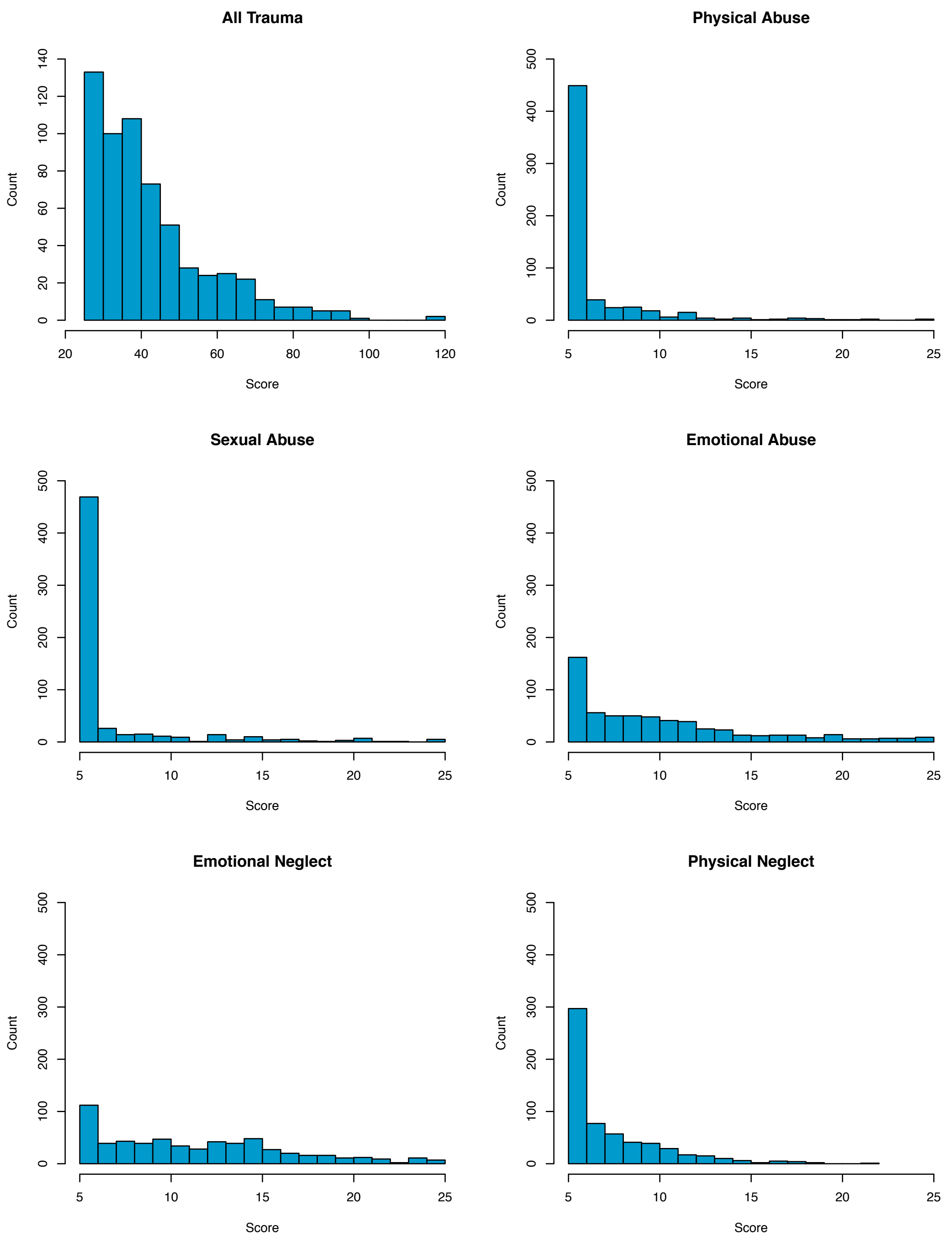

Supplementary Figure 7. Trauma distribution for all patients (n=602). The trauma distribution for all trauma is presented in the first histogram, and distributions for the five subtypes of trauma (Physical Abuse, Sexual Abuse, Emotional Abuse, Emotional Neglect and Physical Neglect) are shown in the other histograms. Trauma score is presented on the x-axis and the count on the y-axis.

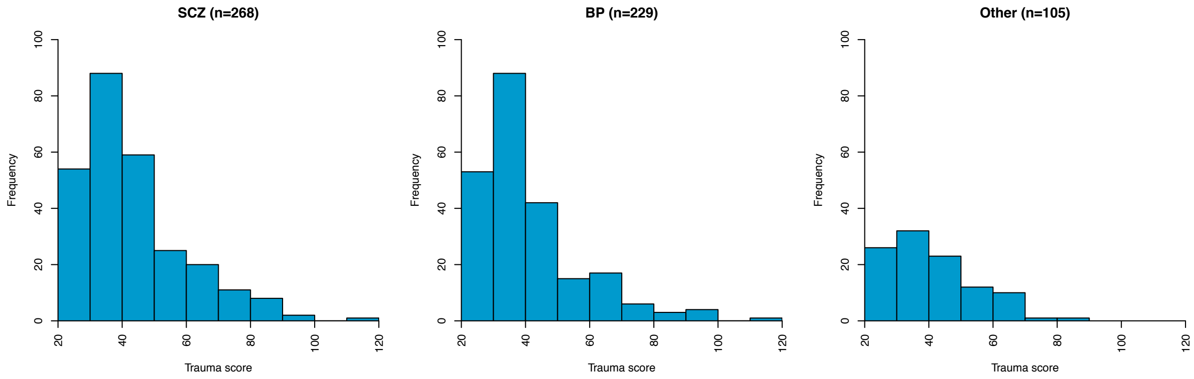

Supplementary Figure 8. Distribution of trauma scores per diagnostic group. Histograms are displayed for the SCZ-group (Schizophrenia group), BP-group (Bipolar group) and the other-group (other type of mental disorder with psychotic features). The total trauma score (obtained from the CTQ-SF) is presented on the x-axis in a scoring range from 25-120, where 120 is the highest possible score. Frequency is presented on the y-axis. The plots show that the distribution of trauma score between the groups is somewhat similar.
